# Correction: Environmental Statistics and Optimal Regulation

**DOI:** 10.1371/journal.pcbi.1003978

**Published:** 2014-10-27

**Authors:** The PLOS Computational Biology Staff

## Notice of Republication

This article was republished on October 10, 2014, to correct errors in the formatting of the equations and paragraphs. These errors were introduced during the typesetting process. Please download this article again to view the correct version. The originally published, uncorrected article and the republished, corrected article are provided here for reference.

## Supporting Information

File S1 Originally published, uncorrected article.
(PDF)

File S2 Republished corrected article.
(PDF)
