# Supplementary material for: Environmental Statistics and Optimal Regulation
Source: PLoS Comput Biol. 2014 Sep 25;10(9):e1003826. doi: 10.1371/journal.pcbi.1003826 (PMC4177669; doi:10.1371/journal.pcbi.1003826)
Supplement: Figure S1 — Optimal regulatory strategy varies with environmental variability and measurement imprecision. Blue curves plot optimal regulatory strategy as a function of cellular readout , for bimodal environments of varying mode width (depicted in leftmost column) and for varying measurement imprecision (depicted in upper row). Black dashed boxes indicate the selected strategies shown in Fig. 4. (PDF) [file pcbi.1003826.s001.pdf]

Environmental statistics and optimal regulation

David A. Sivak* and Matt Thomson*

Supplementary Figure S1

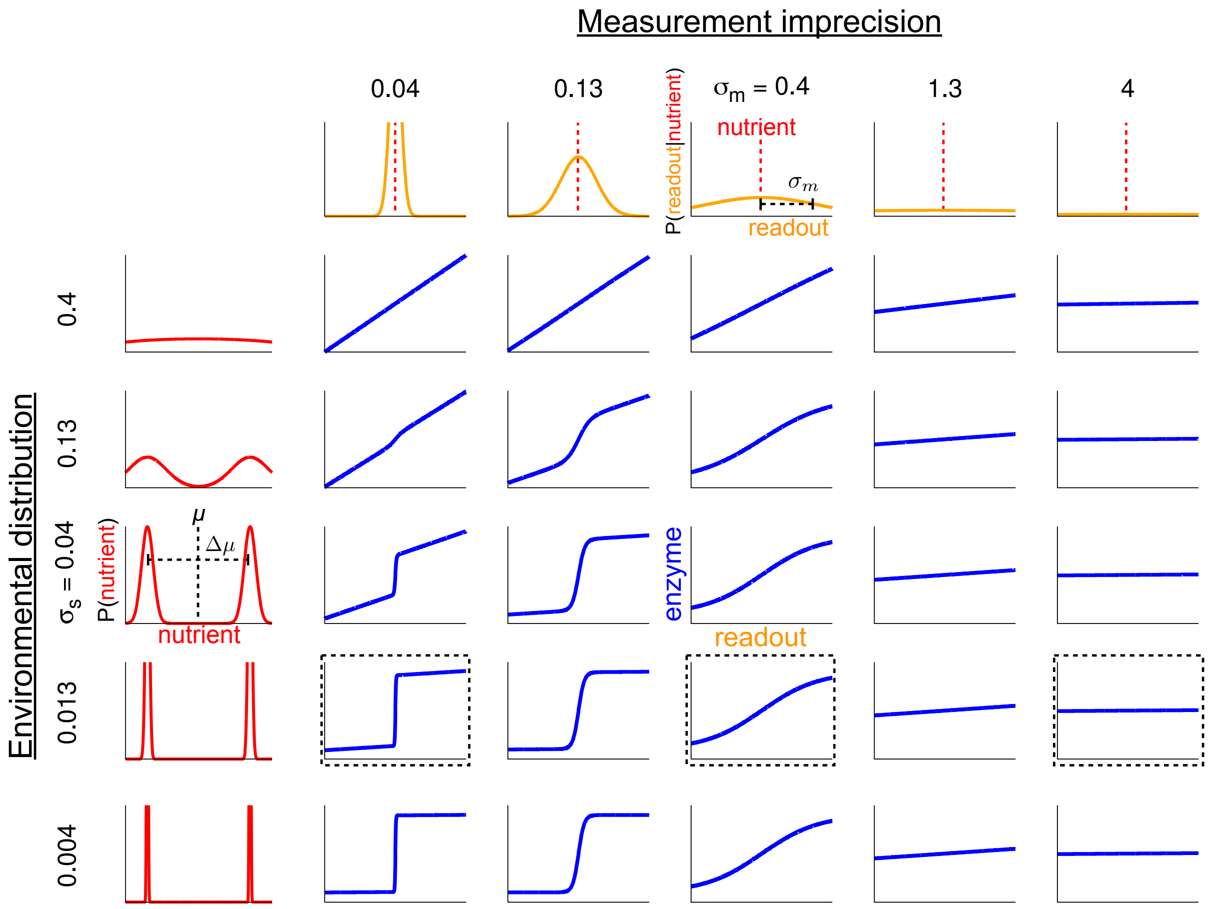

Figure S1. Optimal regulatory strategy varies with environmental variability and measurement imprecision. Blue curves plot optimal regulatory strategy as a function of cellular readout s^*, for bimodal environments of varying mode width (depicted in leftmost column) and for varying measurement imprecision (depicted in upper row). Black dashed boxes indicate the selected strategies shown in Fig. 4.
