# Supplementary material for: Environmental Statistics and Optimal Regulation
Source: PLoS Comput Biol. 2014 Sep 25;10(9):e1003826. doi: 10.1371/journal.pcbi.1003826 (PMC4177669; doi:10.1371/journal.pcbi.1003826)
Supplement: Figure S2 — Optimal regulatory strategy varies with environmental persistence and relative measurement imprecision. Heat maps plot optimal regulatory strategy as a function of present readout (-axis) and past readout (-axis), for varying environmental variability and measurement precision (both depicted in leftmost column) and for varying environmental persistence. Environmental persistence is depicted in upper row as the probability distribution of present nutrient concentration, given steady-state mean (black dashed line) and previous nutrient concentration (red dashed line). Black dashed boxes indicate the selected strategies shown in Fig. 5. (PDF) [file pcbi.1003826.s002.pdf]

# Environmental statistics and optimal regulation

David A. Sivak* and Matt Thomson*

## Supplementary Figure S2

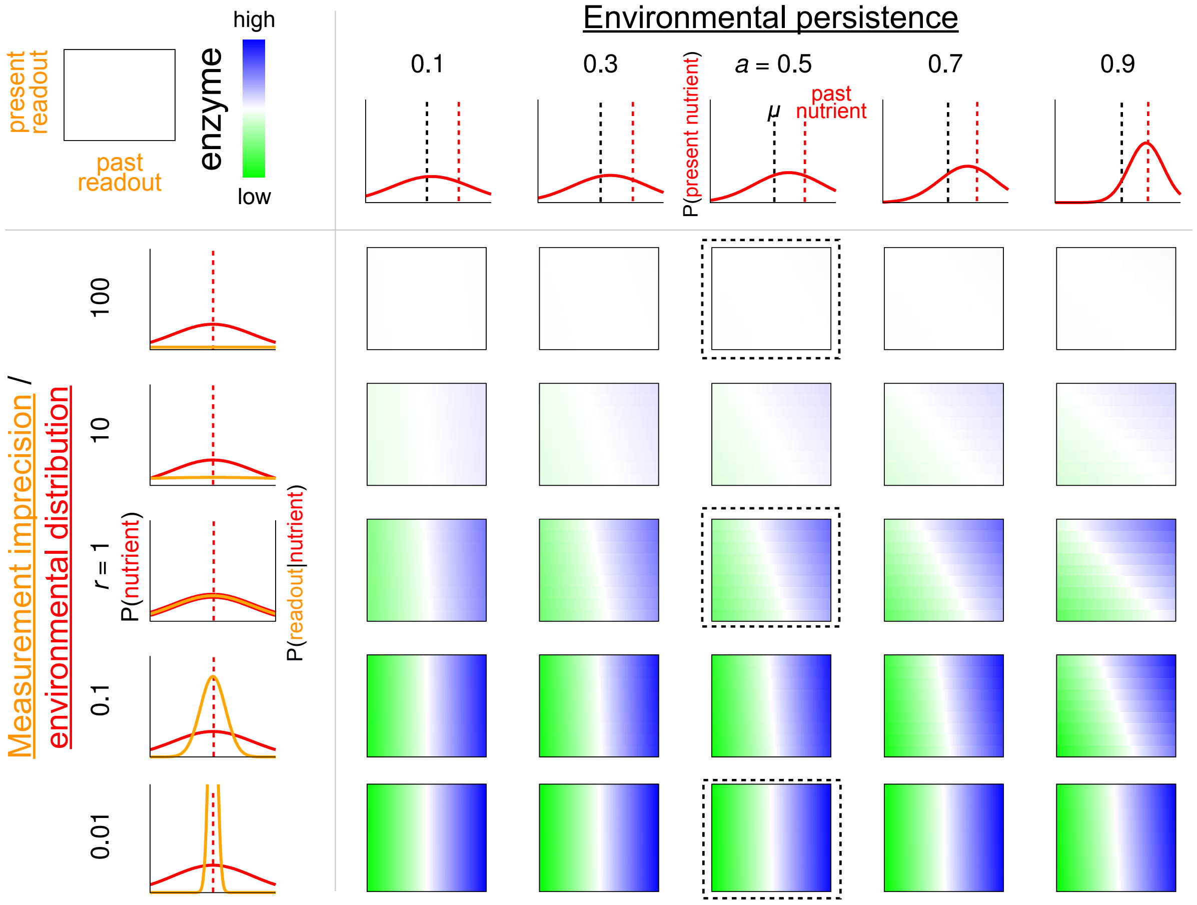


**Figure S2. Optimal regulatory strategy varies with environmental persistence and relative measurement imprecision.** Heat maps plot optimal regulatory strategy as a function of present readout $s_\ell^*$ ($x$-axis) and past readout $s_{\ell-1}^*$ ($y$-axis), for varying environmental variability and measurement precision (both depicted in leftmost column) and for varying environmental persistence. Environmental persistence is depicted in upper row as the probability distribution of present nutrient concentration, given steady-state mean $\mu$ (black dashed line) and previous nutrient concentration (red dashed line). Black dashed boxes indicate the selected strategies shown in Fig. 5.
